# A phase 2 randomized controlled trial of luvadaxistat in treatment of adults with cognitive impairment associated with schizophrenia: results from the ERUDITE study

**DOI:** 10.1038/s41386-026-02410-5

**Published:** 2026-04-24

**Authors:** Ni A. Khin, Reuben H. Fan, Tingting Ge, Hans S. Klein, Jacob S. Ballon, Satjit Brar, Philip D. Harvey, Joshua T. Kantrowitz, Richard S. E. Keefe, Mahnaz Asgharnejad, Venkatesha Murthy, Eiry Roberts, Jaskaran B. Singh

**Affiliations:** 1 Neurocrine Biosciences, Inc., San Diego, CA USA; 2 Department of Psychiatry and Behavioral Sciences, Stanford University, Palo Alto, CA USA; 3 Division of Psychiatry, University of Miami Miller School of Medicine, Miami, FL USA; 4 New York State Psychiatric Institute, New York, NY USA; 5 Columbia University, College of Physicians and Surgeons, New York, NY USA; 6 The Nathan Kline Institute for Psychiatric Research, Orangeburg, NY USA; 7 Duke University Medical Center, Durham, NC USA; 8 Takeda Development Center Americas, Inc., Cambridge, MA USA

**Keywords:** Outcomes research, Schizophrenia

## Abstract

The phase 2 ERUDITE (NCT05182476) study evaluated the efficacy and safety of luvadaxistat, a selective ᴅ-amino acid oxidase inhibitor, for cognitive impairment associated with schizophrenia. ERUDITE was a randomized, double-blind, placebo-controlled, parallel-group study in adults with schizophrenia receiving background antipsychotic therapy. Following a 2-week placebo run-in, participants were randomized 2:1:1 to receive placebo, luvadaxistat 20 mg, or luvadaxistat 50 mg once daily for a 12-week treatment period. The primary endpoint was change from baseline to Day 98 in the Brief Assessment of Cognition in Schizophrenia (BACS) composite score. Safety outcomes included the incidence of treatment-emergent adverse events (TEAEs). Overall, 203 participants were randomized (placebo, *n* = 101; luvadaxistat 20 mg, *n* = 50; luvadaxistat 50 mg, *n* = 52), and 178 (87.7%) completed the treatment period. Luvadaxistat did not significantly improve performance (change from baseline) in BACS composite score versus placebo at Day 98 (least-squares mean difference [95% confidence interval]: 20 mg, –0.7 [–2.8, 1.4], *p* = 0.75; 50 mg, –0.5 [–2.7, 1.6], *p* = 0.69). TEAEs were reported in approximately one-third of participants across groups, with comparable rates observed between the luvadaxistat and placebo groups. TEAEs were mostly mild or moderate, and no safety concerns were identified. Luvadaxistat 20 mg or 50 mg did not show statistically significant changes in cognitive performance or functioning within the ERUDITE study. Trial registration: ClinicalTrials.gov identifier NCT05182476.

## Introduction

Cognitive impairment associated with schizophrenia (CIAS) is a core feature of schizophrenia and refers to deficits in speed of processing, attention, memory, and executive function [1–3]. CIAS is a major contributor to poor functional outcomes, including difficulties in independent living, employment, and social relationships; thus, it is considered a strong predictor of long-term functional disability in individuals with schizophrenia [1–5]. There are no currently approved pharmacological treatments for CIAS, which appears to remain as an unmet need in the management of schizophrenia [1–3].

Glutamatergic signaling deficits, particularly *N*-methyl-d-aspartate (NMDA) receptor hypofunction, are hypothesized to contribute to CIAS pathophysiology [6–8]. Excitotoxic cell death resulting from NMDA receptor overactivation and excessive calcium influx precludes direct NMDA stimulation as a therapeutic option for CIAS [9]. A safer alternative involves enhancing NMDA receptor function indirectly via modulation of NMDA co-agonists, such as ᴅ-serine, at the glycine site [9]. In the hippocampus – which includes subfields such as the dentate gyrus and cornu ammonis 1 and 3, all of which have been implicated in the pathophysiology of schizophrenia [10, 11] – ᴅ-serine is the main co-agonist for certain synaptic NMDA receptors [12]. Luvadaxistat (NBI-1065844/TAK-831), a ᴅ-amino acid oxidase (DAAO) inhibitor, modulates glutamatergic neurotransmission by elevating levels of ᴅ-serine. Using a DAAO inhibitor would minimize the theoretical risks of nephrotoxicity associated with ᴅ-serine treatment; however, limited brain penetration remains a challenge [13]. Luvadaxistat has been shown to increase brain d-serine availability by inhibiting the DAAO enzyme from converting ᴅ-serine to ʟ-serine. [14]. As reviewed in 2015 and 2021 [15, 16], ᴅ-serine has been shown to improve CIAS overall [17] and to improve negative symptoms of schizophrenia in a dose-dependent manner [18], particularly when combined with cognitive training [19, 20].

Preclinical rodent studies have shown that luvadaxistat dose-dependently increases ᴅ-serine levels in plasma, cerebrospinal fluid, and brain tissue [10, 17]; these studies also indicated improved performance in learning and memory tasks, which included novel object recognition, attentional set shifting, and eye-blink conditioning [14, 18]. However, these improvements did not increase monotonically or plateau with higher doses of luvadaxistat; rather, performance declined at sufficiently high exposure to luvadaxistat [14, 18]. In humans, luvadaxistat elevated ᴅ-serine concentrations in plasma and cerebrospinal fluid after single (10–1200 mg) and multiple (15–1200 mg) daily dosing in phase 1 studies [21] and showed near-complete enzyme occupancy at 100–500 mg in a positron emission tomography study (ClinicalTrials.gov: NCT02716987). Furthermore, pharmacokinetic and pharmacodynamic modeling have demonstrated that luvadaxistat 50 mg administered orally results in a predicted enzyme occupancy of 50–90% in the first 12 h after dosing [22]. In a phase 2 study of individuals with schizophrenia (ClinicalTrials.gov: NCT03359785), oral luvadaxistat 50 mg, but not 500 mg, improved mismatch negativity, a cognitive biomarker that is dependent on NMDA receptor function; this indicates that lower doses (i.e., those that would not reach full occupancy) might be more advantageous for improving cognitive performance than higher doses [23].

The phase 2 INTERACT trial (ClinicalTrials.gov: NCT03382639) examined the efficacy and safety of three doses (50, 125, and 500 mg) of daily luvadaxistat versus placebo in adults with schizophrenia [13] and failed to show any significant difference in severity of negative symptoms of schizophrenia. However, luvadaxistat 50 mg showed preliminary signals of improved cognitive performance and functional capacity compared with placebo, as measured by the Brief Assessment of Cognition in Schizophrenia (BACS) and the Schizophrenia Cognition Rating Scale (SCoRS). Furthermore, luvadaxistat was well tolerated at all doses investigated, supporting further evaluation as a treatment for cognitive impairment [13]. The ERUDITE study (ClinicalTrials.gov: NCT05182476) examined the efficacy and safety of luvadaxistat as a targeted treatment for CIAS to determine whether the cognitive benefits observed with luvadaxistat in INTERACT could be replicated.

## Methods

### Study design

**Fig. 1:**
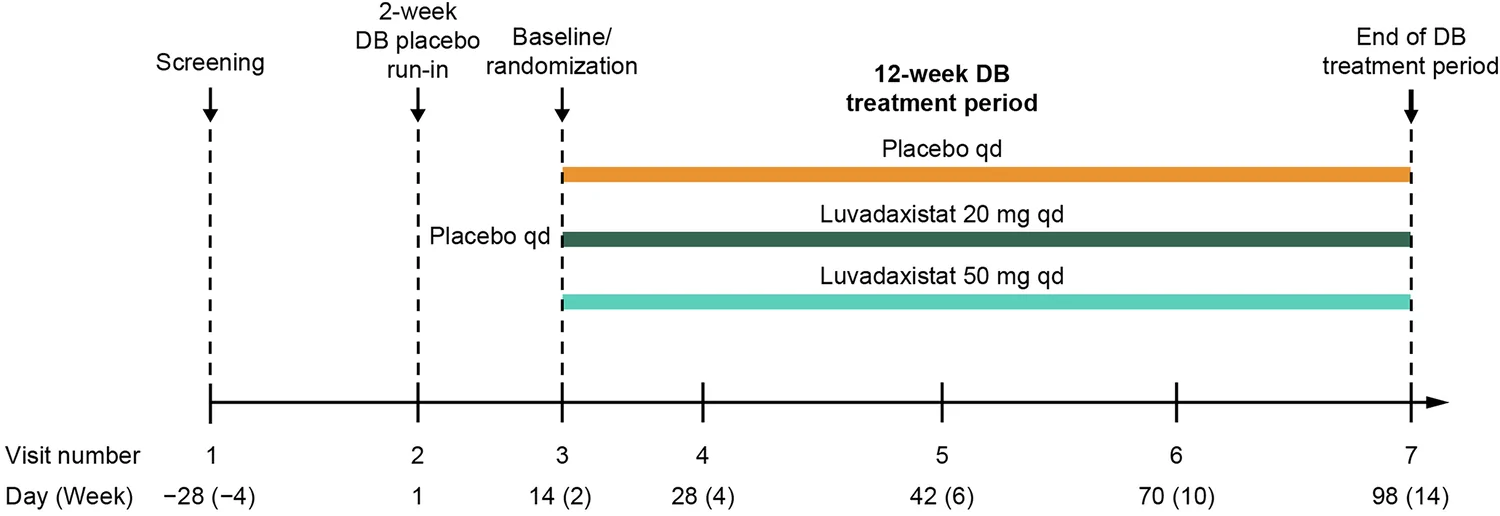
Study design (ERUDITE). DB double-blind, qd once daily.

ERUDITE was a phase 2, randomized, double-blind, placebo-controlled, parallel-group study with an open label extension. The study was designed to evaluate the efficacy, safety, and tolerability of luvadaxistat (NBI-1065844; formerly TAK-831) on improving symptoms of CIAS in adults with schizophrenia who were receiving stable antipsychotic therapy (Fig. 1). The study was conducted across 55 sites in Europe (Bulgaria, Czech Republic, Serbia, Spain) and the USA between December 2, 2021 (date of first informed consent) and October 16, 2024 (date of last patient’s last visit). After a 4-week screening period, participants underwent a 2-week, double-blind placebo run-in to evaluate compliance with study treatment and stability of schizophrenia symptoms. At Day 14, eligible participants started the 12-week double-blind treatment period and were randomized in a 2:1:1 ratio to receive once-daily placebo, luvadaxistat 20 mg, or luvadaxistat 50 mg, administered orally. A greater proportion of participants were randomized to placebo than luvadaxistat to help to reduce the risk of potential nonspecific effects resulting from treatment expectations. Blinding to study treatment was maintained for participants, informants, investigators, site staff, and sponsor study personnel (except supply chain staff) during the placebo run-in and the treatment period.

Randomization was conducted via an interactive web response system and was stratified by age at screening (<35 years vs ≥35 years), and region (USA vs non-USA). Inclusion into the efficacy analysis set (EAS) required participants to demonstrate study treatment compliance (defined as ≥75% adherence between Days 1 and 14 [Visits 2 and 3], based on tablet count, a digital medication adherence app, and an investigator interview) and schizophrenia symptom stability (defined as Positive and Negative Syndrome Scale [PANSS] total scores within 85–115% of the mean across Days −28 to 14 [Visits 1–3]). A 6- or 12-month open-label extension (depending on protocol amendment version) followed the double-blind treatment period, during which all participants received luvadaxistat 50 mg. Safety data from the extension phase will be reported separately.

The study was conducted in accordance with the International Council for Harmonisation Good Clinical Practice guidelines, the Declaration of Helsinki, and applicable local laws and regulations. The protocol, amendments, and informed consent forms were approved by each site’s institutional review board or independent ethics committee, and relevant national health authorities before study initiation.

### Study participants

Eligible participants were adults aged 18–50 years who had received a diagnosis of schizophrenia (per the Diagnostic and Statistical Manual of Mental Disorders, Fifth Edition; as confirmed by the Mini International Neuropsychiatric Interview [MINI] 7.0.2 [24]) at least 1 year before screening, with stable symptoms for at least 3 months before screening. Participants had been receiving psychotropic drugs on a stable regimen for at least 2 months, with no dose increases and less than 25% dose reduction for tolerability. If receiving long-acting injectables (LAIs), no dose change was allowed in the 8 weeks before screening or during the screening period. The target dose range for background antipsychotic therapy was a total daily dose of 2–6 mg of risperidone equivalents; screening antipsychotic medication plasma levels had to be confirmed detectable before baseline. Participants were required to have no more than moderate symptom severity (score ≤4) on PANSS items P1–P6 (delusions, conceptual disorganization, hallucinatory behavior, excitement, grandiosity, suspiciousness) and G9 (unusual thought content) at screening and on Day 1. Participants were also required to have been educated up to a minimum of US eighth grade or equivalent in country of residence and, in the opinion of the investigator, to have sufficient hearing, vision, and cognitive capacity to comply with study procedures. Additionally, participants were required to have a reliable informant (e.g,. a family member, social worker) who could provide additional information to support study assessments and to provide feedback on functioning, if necessary.

Key exclusion criteria included: clinically significant extrapyramidal symptoms (a modified Simpson Angus Scale score >6, excluding item 10); a schizophrenia diagnosis before the age of 12 years; lifetime diagnosis of schizoaffective disorder, bipolar disorder, or obsessive–compulsive disorder; and a recent history of panic disorder, depressive episode within 6 months of screening, or moderate to severe substance use disorder (excluding nicotine dependence) in the 12 months before screening. Participants were also excluded if they had a Calgary Depression Scale for Schizophrenia score of at least 11 or they had any suicidal ideation or behavior in the 6 months before screening or Day 1, clinically significant medical or laboratory abnormalities, intellectual disability, unstable housing, or recent participation in cognitive remediation treatment.

All participants and their informants provided written informed consent after they had received a full explanation of the study.

### Assessments

A detailed schedule of study assessments is shown in Table S1. Cognitive performance and functional capacity assessments included the BACS (primary efficacy), SCoRS (key secondary efficacy), and Virtual Reality Functional Capacity Assessment Tool (VRFCAT); these are further described in Table S2. Clinical assessments included the PANSS and Clinical Global Impression-Severity (CGI-S) scale. All cognitive and clinical assessments were conducted by trained assessors on Days 1, 14, 42, and 98. Efforts were made to perform cognitive assessments at approximately the same time of day by the same assessor throughout the double-blind treatment period to reduce variability resulting from external factors, such as time of day or tester variance [25]. The use of concomitant anticholinergic medications (e.g., benztropine, biperiden) was allowed in the study; however, there were restrictions on daily dose (benztropine equivalent dose maximum of 3 mg/day) and timing of administration (not within 6 h before efficacy assessments). The most recent dose and timing of administration prior to study assessments were recorded at each study visit.

Safety assessments included treatment-emergent adverse events (TEAEs), evaluation of clinical laboratory tests, vital signs, 12-lead electrocardiograms, and the Columbia-Suicide Severity Rating Scale (C-SSRS).

For all efficacy and safety analyses, baseline was defined as the value recorded at the randomization Visit (Day 14). If the Day 14 value was missing, the last available pre-randomization value was used as baseline.

### Endpoints

The primary efficacy endpoint was the change from baseline to Day 98 in the BACS composite score, which indexes overall performance across multiple cognitive domains [26]. The key secondary efficacy endpoint was the change from baseline to Day 98 in the SCoRS interviewer total score, a measure of the impact of cognitive deficits on daily functioning [27]. Additional secondary endpoints included the change from baseline to Day 98 in the VRFCAT score, a measure of functional capacity [28], and the change from baseline to Day 98 in the CGI-S score, a rating scale from 1 to 7 of the overall global severity of schizophrenia. Other endpoints included the change from baseline to Day 98 in PANSS total score, a severity evaluation scale of positive and negative symptoms, as well as general psychopathology items in schizophrenia [29]. Changes from baseline to Day 98 across BACS individual domains were also explored. Safety endpoints included the occurrence of TEAEs and changes from baseline to Day 98 in safety assessments.

### Statistical analysis

Assuming a 25% dropout rate, the study was powered to detect an active treatment effect size of 0.45 at a one-sided α = 0.10 level compared with placebo with 80% power, based on a final total of 135 participants in the EAS completing the study. The EAS included all randomized participants who demonstrated treatment compliance and stable symptomology across Days −28 to 14 (Visits 1 to 3; PANSS scores at each Visit within 85–115% of the mean PANSS score). The EAS was used to analyze the efficacy endpoints (BACS, SCoRS, VRFCAT, and PANSS). The safety analysis set (SAS) included all randomized participants who received at least one dose of the study drug during the double-blind treatment period. The SAS was used to assess TEAEs and changes in safety measures from baseline to Day 98.

The primary endpoint (BACS) and other efficacy endpoints (SCoRS, VRFCAT, and PANSS) were analyzed using an analysis of covariance model. The model included fixed effects for treatment group (placebo, luvadaxistat 20 mg, and luvadaxistat 50 mg), age group (<35 years vs ≥35 years), and region (USA vs non-USA), with baseline score as a covariate. Missing data for primary and key secondary endpoints were handled via multiple imputation, using data from the placebo group (control-based imputation). For the CGI-S, change from baseline to Day 98 was summarized descriptively and analyzed using a Cochran–Mantel–Haenszel test stratified by age and region.

No adjustment for multiplicity was made across the two luvadaxistat dose groups or between the primary and secondary endpoints. As such, all *p* values were reported as nominal.

## Results

### Study population

**Fig. 2:**
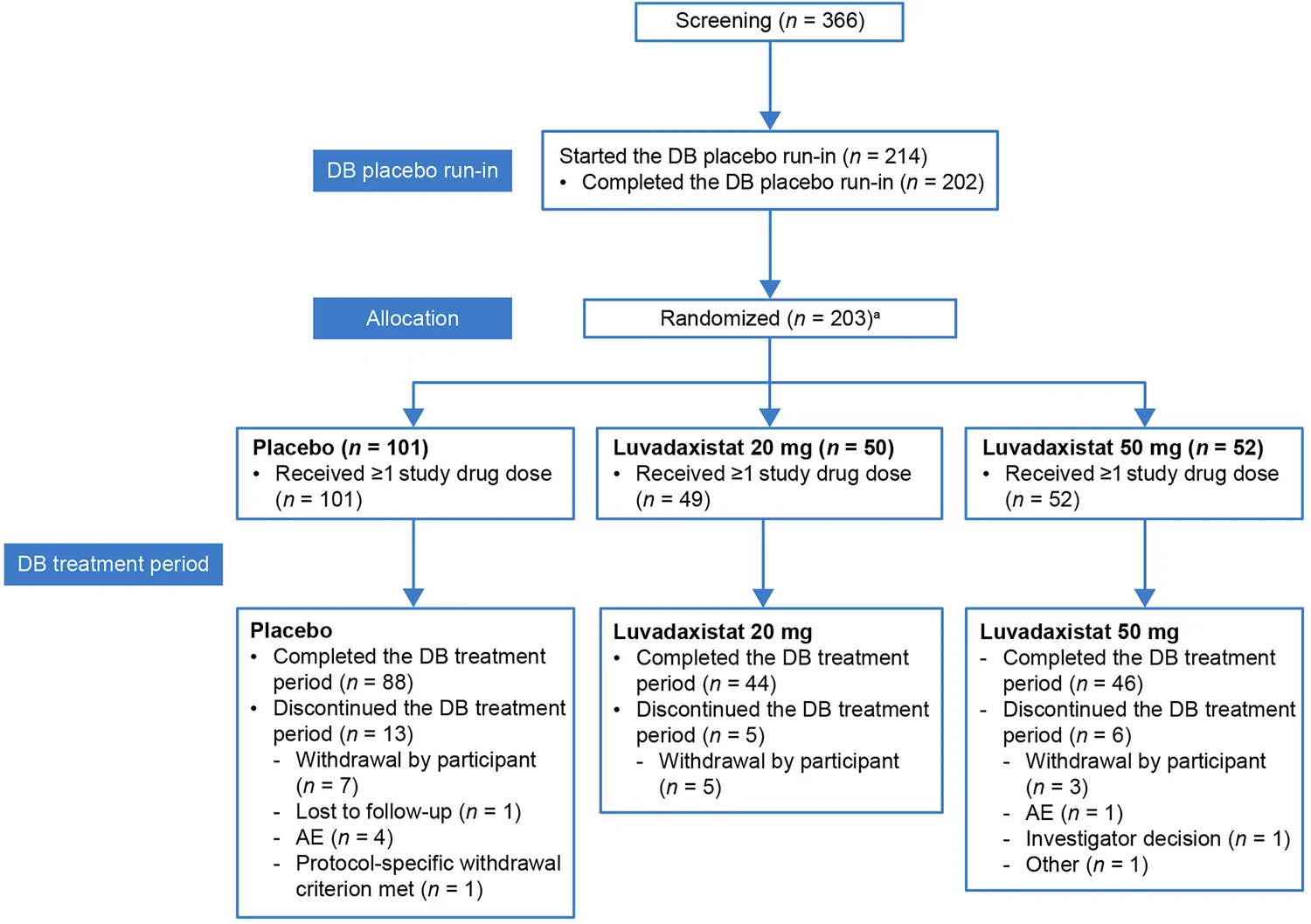
Participant disposition. ^a^One participant was erroneously randomized to the luvadaxistat 20 mg treatment group but did not receive a dose of the study drug after randomization. AE adverse event, DB double-blind.

Of the 366 participants screened, 214 eligible participants were enrolled in the initial 2 weeks of the blinded placebo run-in: 203 were randomized to a treatment group (placebo: *n* = 101; luvadaxistat 20 mg: *n* = 50; luvadaxistat 50 mg: *n* = 52); one participant randomized to luvadaxistat 20 mg did not receive study treatment, and 178 (87.7%) completed the double-blind treatment period (Fig. 2). The EAS included 173 participants (85.2%) (placebo: *n* = 88; luvadaxistat 20 mg: *n* = 42; luvadaxistat 50 mg: *n* = 43), with 161 of these participants completing the study. The SAS included 202 participants (99.5%) (placebo: *n* = 101; luvadaxistat 20 mg: *n* = 49; luvadaxistat 50 mg: *n* = 52).

Demographics and baseline clinical/disease characteristics for the EAS are summarized in Table 1. Overall, the mean (standard deviation [SD]) age was 36.9 (8.1) years; the majority of participants were male (*n* = 111; 64.2%) and White (*n* = 122; 70.5%). Mean (SD) baseline PANSS total score was 61.7 (12.1), and mean (SD) CGI-S score was 3.5 (0.8). Baseline demographics were generally similar across groups, except for sex, for which there was a higher proportion of men in the luvadaxistat 20 mg (71.4%) and 50 mg (69.8%) groups than in the placebo group (58.0%) (Table 1). Aside from this difference, baseline clinical characteristics, including mean PANSS total and CGI-S scores, were similar across the three treatment groups (Table 1). At baseline, mean (SD) BACS composite scores were 32.8 (13.0), 34.1 (15.3), and 36.0 (12.2) in the placebo, luvadaxistat 20 mg, and luvadaxistat 50 mg groups, respectively (Table 1). Mean (SD) SCoRS interviewer total scores were 33.3 (8.7), 34.5 (9.0), and 37.0 (9.7), and mean (SD) VRFCAT scores were 35.6 (16.7), 37.1 (17.6), and 39.7 (18.2), respectively (Table 1). Demographics and baseline clinical/disease characteristics based on the SAS are summarized in Table S3.

**Table 1:** Demographics and baseline clinical/disease characteristics in the EAS.

	Placebo (*n* = 88)	Luvadaxistat 20 mg (*n* = 42)	Luvadaxistat 50 mg (*n* = 43)
Age, years			
Mean (SD)	36.4 (7.7)	37.0 (8.2)	37.6 (9.1)
Min, max	20, 50	18, 50	19, 50
Age category, *n* (%)			
18 years to <35 years	33 (37.5)	16 (38.1)	16 (37.2)
35 years to 50 years	55 (62.5)	26 (61.9)	27 (62.8)
Sex, *n* (%)			
Male	51 (58.0)	30 (71.4)	30 (69.8)
Female	37 (42.0)	12 (28.6)	13 (30.2)
Ethnicity, *n* (%)			
Hispanic or Latino	18 (20.5)	6 (14.3)	7 (16.3)
Not Hispanic or Latino	70 (79.5)	36 (85.7)	36 (83.7)
Race, *n* (%)			
White	62 (70.5)	28 (66.7)	32 (74.4)
Black or African American	19 (21.6)	13 (31.0)	8 (18.6)
Asian	2 (2.3)	1 (2.4)	1 (2.3)
Other	5 (5.7)	0	1 (2.3)
Multiple	0	0	1 (2.3)
Region, *n* (%)			
USA	44 (50.0)	22 (52.4)	23 (53.5)
Non-USA	44 (50.0)	20 (47.6)	20 (46.5)
Weight, kg			
Mean (SD)	88.9 (20.2)	89.0 (18.8)	96.6 (22.4)
Min, max	50.6, 136.4	53.3, 133.0	46.0, 136.0
BMI, kg/m^2^			
Mean (SD)	29.9 (6.2)	29.8 (5.8)	31.4 (7.1)
Min, max	18.4, 43.9	19.3, 39.2	18.2, 43.7
Concomitant anticholinergic medications, *n* (%)			
Yes	11 (12.5)	6 (14.3)	7 (16.3)
No	77 (87.5)	36 (85.7)	36 (83.7)
Background antipsychotic with high anticholinergic burden, *n* (%)			
Yes	29 (33.0)	10 (23.8)	16 (37.2)
No	59 (67.0)	32 (76.2)	27 (62.8)
Number of concomitant antipsychotic medications, *n* (%)			
1	80 (90.9)	41 (97.6)	40 (93.0)
>1	8 (9.1)	1 (2.4)	3 (7.0)
Concomitant antipsychotic medication formulation, *n* (%)			
Oral	68 (77.3)	24 (57.1)	33 (76.7)
LAI	24 (27.3)	18 (42.9)	11 (25.6)
Paliperidone LAI	9 (10.2)	9 (21.4)	5 (11.6)
BACS composite score			
Mean (SD)	32.8 (13.0)	34.1 (15.3)	36.0 (12.2)
Min, max	1, 60	−3, 55	7, 68
SCoRS interviewer total score			
Mean (SD)	33.3 (8.7)	34.5 (9.0)	37.0 (9.7)
Min, max	19, 61	20, 53	20, 61
VRFCAT score			
Mean (SD)	35.6 (16.7)	37.1 (17.6)	39.7 (18.2)
Min, max	−30, 64	−15, 59	−7, 62
CGI-S score			
Mean (SD)	3.5 (0.8)	3.7 (0.7)^a^	3.5 (0.7)
Min, max	1, 6	2, 5	2, 5
PANSS total score			
Mean (SD)	60.8 (12.1)	62.6 (12.1)	62.7 (12.3)
Min, max	33, 98	40, 89	34, 84

EAS: *n* = 173.

*BACS* Brief Assessment of Cognition in Schizophrenia, *BMI* body mass index, *CGI-S* Clinical Global Impression-Severity, *EAS* efficacy analysis set, *LAI* long-acting injectable, *max* maximum, *min* minimum, *PANSS* Positive and Negative Syndrome Scale, *SCoRS* Schizophrenia Cognition Rating Scale, *SD* standard deviation, *VRFCAT* Virtual Reality Functional Capacity Assessment Tool.

^a^CGI-S score data in the luvadaxistat 20 mg group are for 41 individuals.

In the EAS, all participants used concomitant antipsychotic medications, with most using only one concomitant medication (placebo: 90.9%; luvadaxistat 20 mg: 97.6%; luvadaxistat 50 mg: 93.0%) (Table 1). Concomitant anticholinergic medication use was also similar across groups (placebo: 12.5%; luvadaxistat 20 mg: 14.3%; luvadaxistat 50 mg: 16.3%). A background antipsychotic with high anticholinergic burden (as defined by Joshi and colleagues [30]) was reported in 33.0% of placebo, 23.8% of luvadaxistat 20 mg, and 37.2% of luvadaxistat 50 mg participants (Table 1). Oral and long-LAI antipsychotics, respectively, were reported in 77.3% and 27.3% of the placebo group, 57.1% and 42.9% of the luvadaxistat 20 mg group, and 76.7% and 25.6% of the luvadaxistat 50 mg group. The greater proportion of participants in the luvadaxistat 20 mg group receiving an LAI was driven by the increased use of paliperidone LAI (21.4%) relative to the other groups (placebo: 10.2%; luvadaxistat 50 mg: 11.6%) (Table 1). Concomitant antipsychotic medications in the SAS are detailed in Table S4.

### Efficacy

**Fig. 3:**
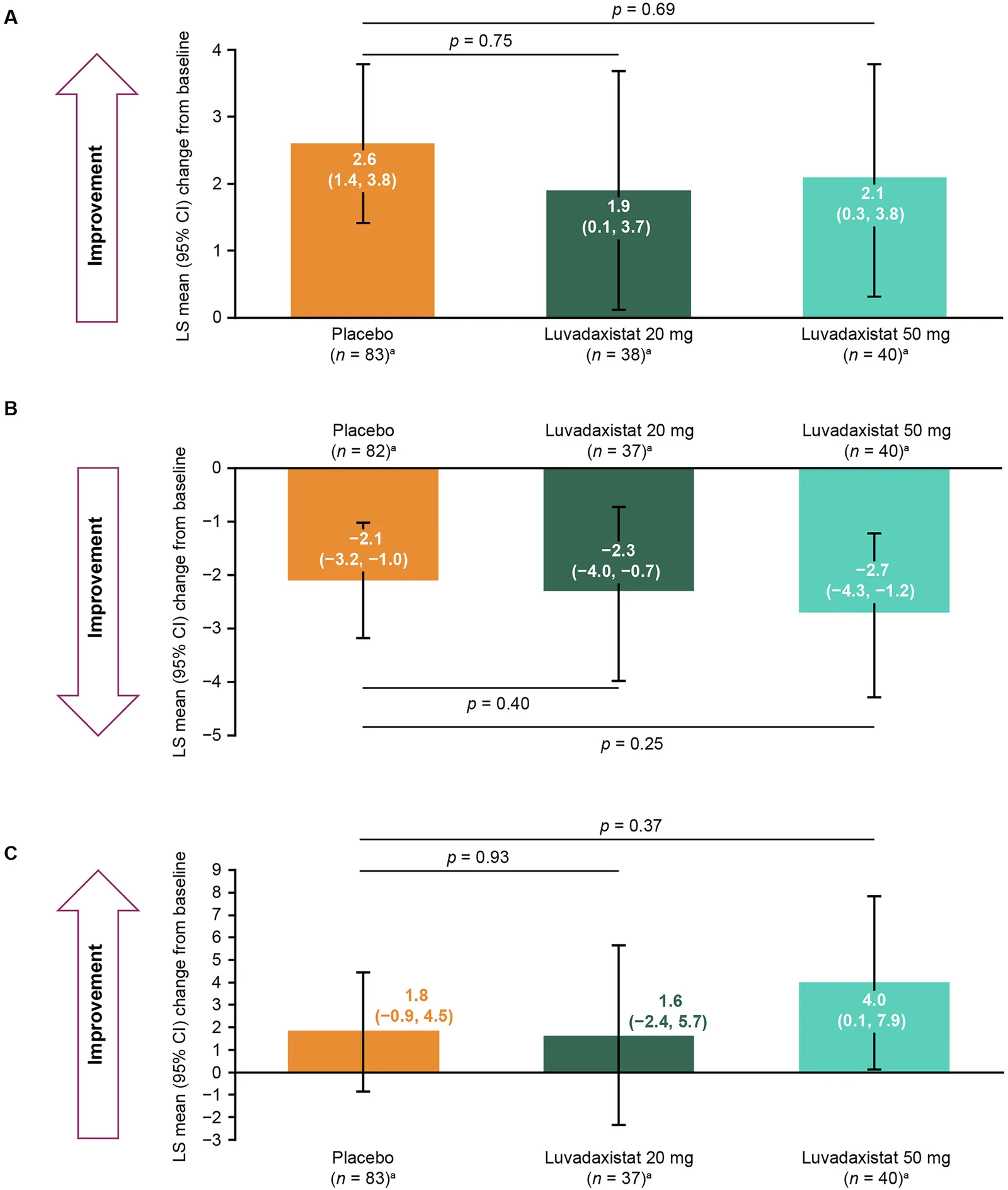
Efficacy endpoints. LS mean change from baseline to Day 98 in BACS composite score (primary endpoint) (**A**), SCoRS interviewer total score (**B**), and VRFCAT score (**C**). EAS: *n* = 173. ^a^Day 98. *p* values (LS mean difference in change from baseline to Day 98, placebo vs luvadaxistat 20 mg and 50 mg). BACS Brief Assessment of Cognition in Schizophrenia, CI confidence interval, EAS efficacy analysis set, LS least-squares, SCoRS Schizophrenia Cognition Rating Scale, VRFCAT Virtual Reality Functional Capacity Assessment Tool.

For the primary endpoint, there was no statistically significant effect on cognitive performance, as assessed by the BACS composite score. The least-squares (LS) mean difference from baseline to Day 98 was −0.7 (95% confidence interval [CI]: −2.8, 1.4; *p* = 0.75) for the luvadaxistat 20 mg treatment group and −0.5 (95% CI: −2.7, 1.6; *p* = 0.69) for the luvadaxistat 50 mg group, compared with the placebo group (Fig. 3A). No statistically significant effects on the individual domains of the BACS assessment were observed (Table S5).

Similarly, compared with placebo, there were no significant changes in ratings of cognitive functioning, based on the SCoRS interviewer total score, with luvadaxistat 20 mg (LS mean difference from baseline to Day 98 [95% CI]: −0.2 [−2.2, 1.7]; *p* = 0.40) or 50 mg (−0.6 [−2.5, 1.2]; *p* = 0.25) (Fig. 3B). Results were consistent for the measure of functional capacity (VRFCAT) for luvadaxistat 20 mg (LS mean difference from baseline to Day 98 [95% CI]: −0.212 [−5.0, 4.6]; *p* = 0.93) and 50 mg (2.1 [−2.6, 6.8]; *p* = 0.37) (Fig. 3C), compared with placebo.

Changes from baseline to Day 98 in CGI-S scores for luvadaxistat 20 mg and 50 mg were not significantly different from those with placebo (*p* = 0.29 and *p* = 0.56, respectively; Table S6).

Compared with placebo, there was no significant difference in overall schizophrenia symptomatology with luvadaxistat 20 mg or 50 mg (LS mean difference in PANSS total score from baseline to Day 98 [95% CI]: −0.6 [−3.3, 2.0]; *p* = 0.64 with 20 mg; 1.9 (−0.7, 4.4); *p* = 0.16 with 50 mg) (Fig. S1).

### Safety and tolerability

Overall, TEAEs were reported in approximately one-third of participants across all treatment groups, with most events being mild or moderate in severity (Table 2). Severe TEAEs were infrequent and occurred only in the placebo and luvadaxistat 50 mg groups. Serious TEAEs were uncommon and occurred in no more than 3% of participants in any group. No deaths were reported in any treatment group. TEAEs leading to discontinuation were reported in four participants receiving placebo and one participant receiving luvadaxistat 50 mg. Frequent TEAEs (occurring in at least three participants in one treatment arm) included somnolence and influenza.

**Table 2:** TEAEs in the SAS.

	Placebo (*n* = 101)	Luvadaxistat 20 mg (*n* = 49)	Luvadaxistat 50 mg (*n* = 52)
≥1 TEAE, *n* (%)	34 (33.7)	13 (26.5)	16 (30.8)
Mild	21 (20.8)	11 (22.4)	12 (23.1)
Moderate	10 (9.9)	2 (4.1)	3 (5.8)
Severe	3 (3.0)	0	1 (1.9)
Serious TEAEs, *n* (%)	3 (3.0)	1 (2.0)	1 (1.9)
Cellulitis	0	1 (2.0)	0
Pneumonia	1 (1.0)	0	0
Urinary tract infection	1 (1.0)	0	0
Seizure	1 (1.0)	0	0
Rash	0	0	1 (1.9)
TEAEs leading to discontinuation, *n* (%)	4 (4.0)	0	1 (1.9)
Proctitis	1 (1.0)	0	0
Upper respiratory tract infection	1 (1.0)	0	0
Urinary tract infection	1 (1.0)	0	0
Lethargy	1 (1.0)	0	0
Seizure	1 (1.0)	0	0
Anxiety	1 (1.0)	0	0
Prostatitis	1 (1.0)	0	0
Rash	0	0	1 (1.9)
Frequent^a^ TEAEs, *n* (%)			
Somnolence	4 (4.0)	0	1 (1.9)
Influenza	4 (4.0)	1 (2.0)	2 (3.8)

SAS: *n* = 202. TEAEs were defined as adverse events occurring after the first dose of randomized study drug and no more than 14 days after the last dose of double-blind study treatment. Events with an unknown onset date were assumed to be TEAEs if they occurred on the first dosing date.

*SAS* safety analysis set, *TEAE* treatment-emergent adverse event.

^a^Frequent TEAEs were those occurring in more than three participants in one treatment arm.

Increases in suicidal ideation at any post-baseline visit, as measured by C-SSRS, were observed in three participants receiving placebo and one participant receiving luvadaxistat 50 mg; no changes from baseline were observed in the luvadaxistat 20 mg group. No major safety signals were identified with respect to the cardiometabolic parameters (routine clinical laboratory values or electrocardiograms). Small increases in QT interval, corrected using Fridericia’s formula, from baseline were observed in a few participants in the luvadaxistat 20 mg and 50 mg groups; however, none exceeded 500 ms, and no participants had clinically significant abnormalities based on investigator electrocardiogram assessment.

## Discussion

There is increasing recognition of the need to address CIAS as a treatment target in patients with schizophrenia [1]. This has driven the development of investigational treatments targeting CIAS, with early preclinical and small-scale clinical trials of NMDA receptor modulators, such as ᴅ-serine, demonstrating preliminary cognitive benefits and further supporting the rationale for more selective agents [15, 16]. As of November 2025, only two compounds, encenicline, an α7 nicotinic acetylcholine receptor agonist, and iclepertin, a glycine transporter-1 inhibitor, have been studied in phase 3 trials, and neither has demonstrated positive results [31, 32].

The present phase 2 study, ERUDITE, similarly failed to replicate the positive cognitive benefits originating from exploratory analyses of luvadaxistat 50 mg in the earlier INTERACT study [13]. During the double-blind treatment period, no significantly different changes were observed for any of the cognitive endpoints (BACS composite score, SCoRS interviewer total score, and VRFCAT score) nor for the clinical endpoints (PANSS total score and CGI-S score) with either luvadaxistat 20 mg or luvadaxistat 50 mg when compared with placebo [13]. The safety and tolerability profile of luvadaxistat in ERUDITE was consistent with that observed in the INTERACT study. Luvadaxistat was generally well tolerated at all doses, with no major safety signals identified across treatment groups.

Although the results did not reveal an impact on cognition within CIAS, the strengths of the study include: the rigorous randomized, double-blind, placebo-controlled study design; inclusion of a placebo run-in to assess symptom stability; and the exclusion of participants receiving unstable antipsychotic medication regimens or those who did not meet adherence thresholds. Well-validated instruments were used for the assessment of cognition, real-world functioning, and clinical symptoms. Further, the observed placebo effect on cognition in ERUDITE (−2.1), measured using SCoRS, was consistent with the 12-week placebo effect on SCoRS observed in another phase 2 study (−2.8) evaluating the efficacy of iclepertin in a similar patient population to that studied in ERUDITE (i.e. adult participants with schizophrenia who were psychiatrically stable without recent symptom exacerbation) [33].

Several limitations should also be considered and further explored to determine the potential impact on observed results within the current study. For example, the current study may have been affected by population heterogeneity and variability in cognitive performance prior to randomization. There were no inclusion/exclusion criteria for the enrichment of participants based on cognitive performance other than investigator judgment. The observed discrepancy across randomized groups in baseline cognition highlights the variability in cognitive performance among participants in the study and may reflect the diagnostic complexity and clinical heterogeneity of schizophrenia, particularly in an international study population. Future trials should consider whether participants should be selected based on levels of pre-baseline cognitive performance, which would be consistent with enrichment strategies already being considered within the field; however, it should be noted that initial cognitive impairment, along with other factors such as age and sex, may not uniformly predict treatment responsiveness [1, 34, 35]. Although unimpaired cognitive performance may potentially suppress treatment effects, it is possible that excessively low cognitive scores could also have an adverse impact on potential treatment benefits [1, 34, 36]. In the current sample, one participant had a baseline BACS score ≥5.0 SD worse than the normative standard (mean: 50, SD: 10) and one participant had a baseline VRFCAT score 8 SD below the normative mean, suggesting some potential assessment challenges. Pre-planned concurrent data oversight throughout the trial did not identify these as invalid scores; however, it remains unclear if schizophrenia without comorbidities would produce such a low performance score. A further consideration is the stability of cognitive performance from screening to baseline, which has been shown to exert an influence on post-baseline performance [36], both for individuals whose scores worsen or improve between screening and baseline. Beyond cognitive performance, the impact of medication adherence on outcomes should also be considered [34]; however, in an exploratory analysis of ERUDITE, participants classified as having high medication adherence did not show significant improvements in cognitive outcomes [37].

Further, generalizability may be limited by the study design. Although a 12-week duration was selected to replicate the timing of cognitive improvements observed in the INTERACT study, it is still unclear how quickly treatment effects may be observed on discrete cognitive ability, as well as on daily functioning. Inclusion criteria, such as required stable antipsychotic treatment and moderate symptom severity, may also inadvertently affect the results. A wide range of antipsychotic treatment was allowed without considering anticholinergic burden, which may have impacted cognitive performance and potential for treatment-related change, given the association between cognitive impairment risk and anticholinergic activity [30]. Moreover, the use of concomitant anticholinergic medications (e.g., benztropine, biperiden) was also allowed in the ERUDITE study with dosing and timing restriction reported prior to each efficacy assessment visit. The frequency of concomitant anticholinergic use was broadly similar across groups. Participants in ERUDITE, like those in INTERACT, were receiving stable antipsychotic treatment during the study, and the relative use of concomitant medications in both studies was broadly comparable [13]. However, in the current study, a greater proportion of participants in the placebo group were taking more than one antipsychotic medication than those in the luvadaxistat groups, who more commonly received LAIs (e.g., paliperidone palmitate) as opposed to daily oral medications. Although not explored within the current trial, differences in concomitant antipsychotic and/or anticholinergic medication use (anticholinergic burden) across treatment groups could potentially have an unintended impact on the outcome measures or could contribute to the overall variability observed within the sample. Additionally, the possibility that site-level management issues (e.g., recruitment density) affected outcomes cannot be excluded [38]. Although missing data were handled using multiple imputation, the potential for bias related to dropout should also be considered.

CIAS is an unmet therapeutic need. It remains critical to reassess and optimize study design elements to help to address the lack of pharmacological intervention for CIAS; these may include the choice of outcome measures, ideal study population characteristics, and statistical considerations when transitioning from early- to late-phase studies [39]. Although development of luvadaxistat has been discontinued, the cross-study insights from all CIAS studies may help to refine future clinical trial designs; these refinements may include participant enrichment based on cognitive performance before the study and/or during the study screening-to-baseline period and mitigating the potential impact of differences in background medication use. Ongoing exploratory analyses from the ERUDITE study may offer additional insights to inform future research approaches targeting CIAS.

## Supplementary information

Supplement

## Data Availability

The data are available following a request to the corresponding author for researchers who provide a methodologically sound proposal. The data will be provided after its de-identification in compliance with applicable privacy laws, data protection, and requirements for consent and anonymization.
