# Supplementary material for: A phase 2 randomized controlled trial of luvadaxistat in treatment of adults with cognitive impairment associated with schizophrenia: results from the ERUDITE study
Source: Neuropsychopharmacology. 2026 Apr 24;51(11):2065–73. doi: 10.1038/s41386-026-02410-5 (PMC13598067; doi:10.1038/s41386-026-02410-5)
Supplement: Supplementary file 1 [file 41386_2026_2410_MOESM1_ESM.docx]

**SUPPLEMENTARY MATERIALS**

**A phase 2 randomized controlled trial of luvadaxistat in treatment of adults with cognitive impairment associated with schizophrenia: results from the ERUDITE study**

Ni A. Khin^1^, Reuben H. Fan^1^, Tingting Ge^1^, Hans S. Klein^1^, Jacob S. Ballon^2^, Satjit Brar^1^, Philip D. Harvey^3^, Joshua T. Kantrowitz^4–6^, Richard S. E. Keefe^7^, Mahnaz Asgharnejad^8^, Venkatesha Murthy^8^, Eiry Roberts^1^ and Jaskaran B. Singh^1^

^1^Neurocrine Biosciences, Inc., San Diego, CA, USA.

^2^Department of Psychiatry and Behavioral Sciences, Stanford University, Palo Alto, CA, USA.

^3^Division of Psychiatry, University of Miami Miller School of Medicine, Miami, FL, USA.

^4^New York State Psychiatric Institute, New York, NY, USA.

^5^Columbia University, College of Physicians and Surgeons, New York, NY, USA.

^6^The Nathan Kline Institute for Psychiatric Research, Orangeburg, NY, USA.

^7^Duke University Medical Center, Durham, NC, USA.

^8^Takeda Development Center Americas, Inc., Cambridge, MA, USA.

**Fig. S1	LS mean change from baseline to Day 98 in PANSS**

EAS: *n* = 173. ^a^Day 98. *p* values (LS mean difference in change from baseline to Day 98, placebo vs luvadaxistat 20 mg and 50 mg). CI, confidence interval; EAS, efficacy analysis set; LS, least-squares; PANSS, Positive and Negative Syndrome Scale.

**Table S1.**	Schedule of study procedures during screening, double-blind placebo run-in, and the double-blind treatment period.

|  | **Screening^a^** | **Double-blind placebo run-in  (2 weeks)** | **Double-blind treatment period (12 weeks)** | | | | |
| --- | --- | --- | --- | --- | --- | --- | --- |
| Study Day(s) | −28 to 0 | 1 | 14 | 28 | 42 | 70 | 98 |
| Week | −4 |  | 2 | 4 | 6 | 10 | 14 |
| Visit number | 1 | 2 | 3 | 4 | 5 | 6 | 7 |
| BACS |  | X | X |  | X |  | X |
| VRFCAT |  | X | X |  | X |  | X |
| SCoRS^b^ |  | X | X |  | X |  | X |
| PANSS^b^ | X | X | X |  | X |  | X |
| CGI-S |  | X |  |  |  |  | X |

The ‘X’ indicated that the assessment was conducted at that visit. ^a^The screening period could be extended by up to 14 days for certain unavoidable circumstances (such as the COVID-19 pandemic) with approval from sponsor or designee after discussion of individual circumstances. Individuals who did not meet antipsychotic medication stability criteria or other entry criteria during the screening period could be considered for rescreening one time with the approval of the sponsor or designee. ^b^Informant interviews were required for the SCoRS and PANSS assessments; informants could provide input by attending on-site visits or via virtual visits up to 3 days before the individual’s visit. BACS, Brief Assessment of Cognition in Schizophrenia; CGI-S, Clinical Global Impression-Severity; PANSS, Positive and Negative Syndrome Scale; SCoRS, Schizophrenia Cognition Rating Scale; VRFCAT, Virtual Reality Functional Capacity Assessment Tool.

**Table S2.**	Cognitive assessments.

| **Cognitive assessment** | **Description** |
| --- | --- |
| **BACS** | The BACS is a validated cognitive test battery designed to evaluate treatment-related changes in cognition across six domains: verbal memory, working memory, motor function, verbal fluency, attention/speed of processing, and executive function. Each subtest has its own raw score range (e.g. 0–75 for verbal memory, 0–225 for verbal fluency). Raw scores are standardized for age and sex to produce a composite score, which serves as a measure of overall cognitive functioning. Higher composite scores indicate better cognitive ability [28]. |
| **SCoRS** | The SCoRS is a validated, interview-based instrument that assesses the impact of cognitive deficits on daily functioning. It includes 20 items, each rated on a 4-point scale, with higher scores reflecting greater impairment. The interviewer total score is derived by summing the ratings for each item. The SCoRS focuses on real-world cognitive functioning across the seven MATRICS cognitive domains [28]. |
| **VRFCAT** | The VRFCAT is a computerized, immersive assessment tool that evaluates functional capacity across four scenarios: searching for ingredients of a grocery list, navigating transport, shopping, and managing currency. The tool measures accuracy and efficiency in completing task objectives. VRFCAT produces a standardized T-score representing relative efficiency compared with a normative, unaffected sample, corrected for age and sex, with average performance at 50 and a standard deviation of 10. Higher scores represent better functional capacity. It takes approximately 20 minutes to complete and is sensitive to functional deficits in schizophrenia [40]. |

BACS, Brief Assessment of Cognition in Schizophrenia; MATRICS; Measurement and Treatment Research to Improve Cognition in Schizophrenia; SCoRS, Schizophrenia Cognition Rating Scale; VRFCAT, Virtual Reality Functional Capacity Assessment Tool.

**Table S3.**	Select demographics and baseline clinical/disease characteristics in the SAS

|  | **All participants**  **(*N* = 202)** |
| --- | --- |
| Age, years  Mean (SD)  Min, max | 36.7 (8.1) 18, 50 |
| Sex, *n* (%)  Male  Female | 127 (62.9)  75 (37.1) |
| Ethnicity, *n* (%)  Hispanic or Latino  Not Hispanic or Latino | 41 (20.3)  161 (79.7) |
| Race, *n* (%)  American Indian or Alaska Native  Asian  Black or African American  White  Other  Multiple | 1 (0.5)  4 (2.0)  48 (23.8)  140 (69.3)  8 (4.0)  1 (0.5) |
| Number of concomitant antipsychotic medications prescribed, *n* (%)  1  >1 | 189 (93.6)  13 (6.4) |
| CGI-S score  Mean (SD)  Min, max | 3.5 (0.7)  2, 5 |
| PANSS total score  Mean (SD)  Min, max | 62.7 (12.3)  34, 84 |

SAS: *n* = 173. CGI-S, Clinical Global Impression-Severity; max, maximum; min, minimum;
PANSS, Positive and Negative Syndrome Scale; SAS, safety analysis set; SD, standard deviation.

**Table S4.**	Concomitant antipsychotic medications in the SAS.

| **Medication, *n* (%)** | **Placebo (*n* = 101)** | **Luvadaxistat  20 mg (*n* = 49)** | **Luvadaxistat  50 mg (*n* = 52)** |
| --- | --- | --- | --- |
| Aripiprazole | 22 (21.8) | 8 (16.3) | 10 (19.2) |
| Olanzapine | 22 (21.8) | 10 (20.4) | 13 (25.0) |
| Aripiprazole LAI^a^ | 14 (13.9) | 8 (16.3) | 3 (5.8) |
| Risperidone | 13 (12.9) | 5 (10.2) | 4 (7.7) |
| Paliperidone LAI^a^ | 10 (9.9) | 10 (20.4) | 7 (13.5) |
| Quetiapine/quetiapine fumarate | 7 (6.9) | 2 (4.1) | 3 (5.8) |
| Cariprazine/cariprazine hydrochloride | 7 (6.9) | 2 (4.1) | 3 (5.8) |
| Paliperidone | 0 (0.0) | 1 (2.0) | 2 (3.8) |
| Amisulpride | 4 (4.0) | 1 (2.0) | 0 (0.0) |
| Haloperidol | 3 (3.0) | 1 (2.0) | 1 (1.9) |
| Lurasidone | 3 (3.0) | 0 (0.0) | 1 (1.9) |
| Olanzapine LAI^a^ | 1 (1.0) | 2 (4.1) | 2 (3.8) |
| Ziprasidone | 1 (1.0) | 1 (2.0) | 0 (0.0) |
| Fluphenazine | 0 (0.0) | 0 (0.0) | 2 (3.8) |
| Haloperidol LAI^a^ | 1 (1.0) | 0 (0.0) | 1 (1.9) |
| Brexpiprazole | 0 (0.0) | 0 (0.0) | 1 (1.9) |
| Asenapine | 0 (0.0) | 0 (0.0) | 1 (1.9) |
| Risperidone LAI^a^ | 0 (0.0) | 0 (0.0) | 1 (1.9) |
| Chlorprothixene | 0 (0.0) | 1 (2.0) | 0 (0.0) |
| Fluphenazine LAI^a^ | 3 (3.0) | 0 (0.0) | 1 (1.9) |

SAS: *n* = 202. ^a^Paliperidone LAI includes paliperidone palmitate; aripiprazole LAI includes aripiprazole lauroxil; olanzapine LAI includes olanzapine embonate; haloperidone LAI include haloperidone decanoate; fluphenazine LAI includes fluphenazine decanoate. LAI, long-acting injectable; SAS, safety analysis set.

**Table S5.**	LS mean treatment differences in changes from baseline to Day 98 across BACS individual domains.

|  | **Placebo** | **Luvadaxistat**  **20 mg** | **Luvadaxistat**  **50 mg** |
| --- | --- | --- | --- |
| *BACS verbal memory domain* | | | |
| n | 84 | 38 | 40 |
| LS mean (95% CI) | 2.0 (0.4, 3.7) | 2.2 (−0.2, 4.7) | 2.3 (−0.1, 4.7) |
| LS mean difference  (95% CI) | - | 0.2 (−2.7, 3.1) | 0.3 (−2.6, 3.2) |
| *p* value | - | 0.4447 | 0.4253 |
| *BACS working memory domain* | | | |
| n | 83 | 37 | 40 |
| LS mean (95% CI) | 1.2 (−0.5, 2.9) | 1.8 (−0.7, 4.4) | 1.3 (−1.1, 3.7) |
| LS mean difference  (95% CI) | - | 0.6 (−2.4, 3.7) | 0.1 (−2.8, 3.0) |
| *p* value | - | 0.3422 | 0.4756 |
| *BACS motor function domain* | | | |
| n | 83 | 38 | 40 |
| LS mean (95% CI) | 1.5 (0.1, 2.9) | 0.7 (−1.4, 2.7) | 1.8 (−0.2, 3.9) |
| LS mean difference  (95% CI) | - | −0.9 (−3.4, 1.6) | 0.3 (−2.2, 2.8) |
| *p* value | - | 0.7532 | 0.4073 |
| *BACS verbal fluency domain* | | | |
| n | 82 | 38 | 40 |
| LS mean (95% CI) | 0.9 (−0.2, 2.1) | −1.0 (−2.7, 0.7) | 0.6 (−1.0, 2.3) |
| LS mean difference  (95% CI) | - | −1.9 (−4.0, 0.1) | −0.3 (−2.3, 1.7) |
| *p* value | - | 0.9699 | 0.6182 |
| *BACS attention/processing speed domain* | | | |
| n | 83 | 38 | 40 |
| LS mean (95% CI) | 3.3 (2.0, 4.6) | 1.9 (0.0, 3.7) | 2.9 (1.1, 4.8) |
| LS mean difference  (95% CI) | - | −1.4 (−3.7, 0.8) | −0.4 (−2.6, 1.9) |
| *p* value | - | 0.8908 | 0.6218 |
| *BACS executive function domain* | | | |
| n | 83 | 38 | 40 |
| LS mean (95% CI) | 2.1 (−0.3, 4.5) | 2.4 (−1.2, 6.0) | 0.8 (−2.8, 4.3) |
| LS mean difference  (95% CI) | - | 0.3 (−4.0, 4.6) | −1.3 (−5.6, 2.9) |
| *p* value | - | 0.4484 | 0.7311 |

LS mean based on ANCOVA, which included fixed effects for treatment group, age group, and region, with baseline score as a covariate; *p* values (LS mean difference in change from baseline to Day 98, luvadaxistat [20 mg and 50 mg] - placebo).

ANCOVA, analysis of covariance; BACS, Brief Assessment of Cognition in Schizophrenia; CI, confidence interval; LS, least-squares.

**Table S6.**	Number and percentage of individuals in CGI-S categories at baseline and at Day 98.

|  | **Placebo (*n* = 88)** | | **Luvadaxistat 20 mg (*n* = 42)** | | **Luvadaxistat 20 mg (*n* = 43)** | |
| --- | --- | --- | --- | --- | --- | --- |
|  | **Baseline** | **Day 98** | **Baseline** | **Day 98** | **Baseline** | **Day 98** |
| *n* | 88 | 84 | 41 | 37 | 43 | 39 |
| CGI-S category, *n* (%) |  |  |  |  |  |  |
| 1 = Normal, not at all ill | 1 (1.1) | 0 | 0 | 0 | 0 | 0 |
| 2 = Borderline ill | 6 (6.8) | 15 (17.9) | 3 (7.3) | 2 (5.4) | 3 (7.0) | 5 (12.8) |
| 3 = Mildly ill | 38 (43.2) | 35 (41.7) | 11 (26.8) | 18 (48.6) | 17 (39.5) | 20 (51.3) |
| 4 = Moderately ill | 39 (44.3) | 32 (38.1) | 24 (58.5) | 15 (40.5) | 20 (46.5) | 14 (35.9) |
| 5 = Markedly ill | 3 (3.4) | 2 (2.4) | 3 (7.3) | 2 (5.4) | 3 (7.0) | 0 |
| 6 = Severely ill | 1 (1.1) | 0 | 0 | 0 | 0 | 0 |
| 7 = Among the most extremely ill | 0 | 0 | 0 | 0 | 0 | 0 |
| *p* value |  |  |  | 0.29 |  | 0.56 |

EAS: *n* = 173. *p* values are from the Cochran–Mantel–Haenszel χ^2^ test adjusted for age group and region. CGI‑S, Clinical Global Impression-Severity; EAS, efficacy analysis set.
